# How Much Instructional Time Is Necessary? Mid-intervention Results of Fundamental Movement Skills Training Within ABA Early Intervention Centers

**DOI:** 10.3389/fnint.2020.00024

**Published:** 2020-05-12

**Authors:** Isabella Theresa Felzer-Kim, Janet Lynn Hauck

**Affiliations:** Department of Kinesiology, Michigan State University, East Lansing, MI, United States

**Keywords:** autism spectrum disorder, motor skills, visual supports, applied behavior analysis, intervention

## Abstract

**Background**: The purpose of this study was to explore the question of the minimal amount of instructional time needed to still be effective by assessing the efficacy at mid-intervention of an early fundamental movement skill (FMS) intervention for preschoolers with Autism Spectrum Disorder (ASD).

**Method**: Fourteen preschoolers participated in this randomized controlled trial daily over 10 weeks (10 h total at mid-intervention). A two-factor mixed MANOVA tested the significance of group*time interactions for two dependent variables: object control and locomotor raw scores on the Test of Gross Motor Development—III.

**Results**: Group*time interactions approached significance with large effect sizes on the vector of both dependent variables and in a univariate fashion on object control scores, but not locomotor scores.

**Conclusions**: These findings hold relevance for physical educators working with young children with ASD, indicating that 10 h of FMS instruction, at least in this form, is not adequate to improve FMS.

## Introduction

Although not part of the diagnostic criteria for Autism Spectrum Disorder (ASD), children with ASD show frequent motor delays (Staples and Reid, 2010) that begin early in life and become more significant with age (Lloyd et al., 2013). It appears that motor skills in this population also relate to developmental areas beyond the physical (MacDonald et al., 2014), emphasizing the importance of fundamental movment skill (FMS) training and adapted physical education (APE) services for children with ASD. This is increasingly relevant for young children, given the strong evidence that early intervention is effective for children with ASD (Reichow et al., 2012).

Early FMS interventions for those with ASD show promising results in research settings. A 12-week FMS intervention implemented by APE researchers improved object manipulation and overall motor scores for nine 4-year-olds with ASD (Bremer et al., 2015). Some individual FMS improved from an intervention orchestrated by APE researchers and a special education teacher within an early intervention classroom with five children aged 3–7 showing ASD-like characteristics (Bremer and Lloyd, 2016). Finally, the locomotor and ball skills of 11 children aged 4–6 (nine controls) improved in a summer-camp motor skill intervention, again implemented by APE researchers (Ketcheson et al., 2017).

FMS interventions for children with ASD show promising results; however, no matter the setting, competing curricular demands will always be present. It is therefore necessary to know how much direct FMS instructional time is required, in order to justify lesson time. In the 2018, Patricia Austin Award Presentation, this question was addressed as part of a meta-analysis (Case, 2018). The findings showed a substantial publication bias, wherein most published interventions showed a significant treatment effect, and very few used less than 12 instructional hours. The issue, it appears, is underreporting of FMS interventions with null results. Thus, the question of minimal instructional time needed is still unanswered.

Early Intensive Behavioral Intervention (EIBI) centers, frequently based on Applied Behavior Analysis (ABA) techniques, have gained attention in recent times. These centers are generally specified for children with severe ASD who qualify for intensive behavioral treatment before entry into kindergarten. There are typically no APE services offered in this environment. The EIBI environment has not been used for early FMS intervention. These centers typically use individualized therapy plans and a small staff to student ratio, thus they hold promise as a delivery platform for early FMS intervention services.

The purpose of this study was to explore this question of minimally effective FMS instruction time within an ecologically valid environment, the EIBI clinic. Baseline data and mid-intervention outcomes are presented here from a 20-week FMS randomized controlled trial. Post-intervention and follow-up results will be published separately upon their collection and analysis.

## Materials and Methods

### Participants

Fourteen children were recruited from two campuses of an ABA EIBI clinic and randomized within each campus to form a control (*n* = 6) and intervention group (*n* = 8; Table 1).

**Table 1:** Comparison of control and intervention group TGMD—III raw scores from baseline to mid-intervention by repeated measures MANOVA, and baseline descriptive information.

	Control (*n* = 6)		Intervention (*n* = 8)		Total (*n* = 14)	
	Mean	SD	Mean	SD	Mean	SD
**TGMD—III**					
Pre-intervention Locomotor	5.000	4.195	4.750	4.683	4.857	4.312
Pre-intervention Ball skills	5.167	3.764	5.625	4.984	5.429	4.345
Pre-intervention Total	10.170	7.574	10.380	8.700	10.290	7.927
Mid-intervention Locomotor	5.800	4.760	8.500	9.040	7.462	7.557
Mid-intervention Ball skills	6.600	3.580	12.250	7.940	10.077	7.017
Mid-intervention Total	12.400	7.700	20.750	16.450	45.000	17.539
**Descriptive information**					
Height (cm)	104.700	7.562	105.260	6.536	105.050	6.390
Weight (kg)	17.167	4.574	18.400	2.662	17.938	3.230
BMI percentile	70.000	39.509	68.875	29.902	69.308	32.284
ADOS-2 CSS	7.500	1.975	8.286	2.059	7.923	1.977
Gender	3F; 3M		1F; 7M		4F; 10M	
Age (months)	53.833	7.167	53.875	7.019	53.857	6.803
Annual Household Income	1 < $24,000		1 < $24,999		2 < $24,999
	2 $50,000–$75,000		1 $25,000–$49,999		1 $25,000–$49,999
	3 missing		1 $50,000–$74,999		3 $50,000–$74,999
			1 > $75,000		1 > $75,000
			4 missing		7 missing
Race	2 White (33.3%)		5 White (62.5%)		7 White (50%)
	3 AA (50.0%)		2 AA (25.0%)		5 AA (35.7%)
	1 Asian (16.7%)		1 Asian (12.5%)		2 Asian (14.3%)

### Procedure

All procedures were approved by an ethical board before data collection began and all participants’ caregivers gave informed consent. Caregivers supplied descriptive characteristics on a questionnaire. Autism Severity (Autism Diagnostic Observation Scales–2 calibrated severity score; ADOS-2) was measured by ABA staff and reported at baseline. Anthropometrics and FMS (Test of Gross Motor Development—III—TGMD—III) were assessed prior to any baseline and following 10 weeks (mid-intervention) of intervention. For anthropometrics, height without shoes was measured to the nearest 2 cm (Seca Stadiometer) and weight in light clothing was measured to the nearest 0.1 kg (standing scale). Body mass index (BMI) percentile was calculated according to Centers for Disease Control growth curves.

### Intervention

Direct FMS instruction sessions lasted 15 min each and occurred 4 days per week for 10 weeks. Each session consisted of discrete trial training in one of the 13 FMS (run, gallop, skip, hop, jump, slide, two-handed strike of a stationary ball, one-handed strike of a self-bounced ball, one handed dribble, kick, two-handed catch, overhand throw, and underhand throw) for one individual child. Trials were implemented by the EIBI behavior technician already working with the child. One research staff was present to answer questions and collect video. Each trial consisted of viewing a tablet-displayed video of the FMS, a picture task card, and an abbreviated verbal direction (Breslin and Rudisill, 2011). Following this stimulus, the participant completed one trial of the skill, with the behavior technician implementing a most-to-least physical prompting hierarchy and providing immediate differential reinforcement (reinforcement following attempts, and more potent reinforcement following correct attempts). An additional 5 min per day, 4 days per week for 10 weeks, the entire intervention group at each campus played rotating active social games without direct instruction in FMS. The control group continued therapy as usual and were simply tested on FMS twice with an interval of 10 weeks.

### Measures

#### TGMD-III

The present study used picture task cards (Breslin and Rudisill, 2011), short standardized instructions (Breslin and Rudisill, 2011), and administration provided by a single live model (Allen et al., 2017). Video-recorded assessments were scored by individuals blinded to group (control or intervention) and time (baseline or mid-intervention), and whom had achieved 90% reliability (Rintala et al., 2017) using videos and scores disseminated by the assessment authors.

### Statistical Analysis

To check equivalence of groups, a MANOVA was conducted to compare the control and intervention groups’ baseline TGMD—III and ADOS-2 scores. A two-factor mixed MANOVA (each subject tested twice in time but belonged to only one group) tested the significance of group*time interactions for TGMD–III raw scores in object control and locomotor subtests from baseline to mid-intervention between the two groups. This allowed tests of the multivariate interaction effects as well as the univariate interaction effects for each dependent variable. The assumptions of linearity of relationships among the dependent variables, multivariate normality, and homogeneity of variance-covariance matrices between groups were tested before conducting the mixed MANOVA, even though sample sizes were not more than 1.5 times different (Leech et al., 2014). Matrix scatterplots and correlation matrices showed linearity but not multicollinearity (*r* < 0.80; Vatecheva et al., 2016). Before beginning any parametric tests, normality was assessed using the Shapiro–Wilk test for normality; the dependent variables did not violate the assumption of normality. Box’s M test was conducted to check the homogeneity assumption, and no significant differences were found between the covariance matrices; Wilk’s Lambda was an appropriate test to use. A significant interaction term would indicate that intervention group TGMD—III scores had improved by mid-intervention, compared to the control. Box-and-whisker plots visually represent these findings and Partial η^2^ were calculated to represent effect sizes. All statistical procedures were carried out in SPSS version 25 (Nie et al., 1970) with a pre-determined alpha of 0.05.

## Results

**Figure 1:**
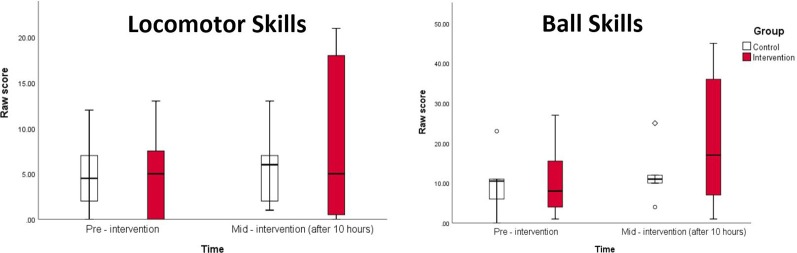
Box-and-whisker plots of total, locomotor, and ball skill raw scores by interventional group at baseline and mid-intervention. Higher scores indicate better motor performance.

No significant differences arose between groups at baseline, indicating successful randomization (see Table 1). The two-factor mixed MANOVA showed an insignificant interaction between time and group for the multivariate vector of the two dependent variables (*F*_(2,10)_ = 2.436; *p* = 0.137; ES = 0.328). For univariate interaction effects, ball skills (*F*_(1,11)_ = 4.640; *p* = 0.054; ES = 0.297) showed a large but insignificant effect size. The interaction term for locomotor skills (*F*_(1,11)_ = 1.232; *p* = 0.291; ES = 0.101) was not significant nor approaching significance, and showed a medium effect size. Table 1 and Figure 1 detail these findings.

## Discussion

Here, 10 h of direct instruction did not alter FMS in this sample. A study in a similar population used 160 h of intervention, and saw improvements in all subtests on the TGMD—II by 40 h (Ketcheson et al., 2017). Another used 27 h of intervention and saw improvements in most children’s catch, roll and strike, run, gallop, jump, and kick; however, the study’s size did not allow for group statistics (Bremer and Lloyd, 2016). Finally, another study saw improvements in object control, but not locomotor or total motor scores after 12 h of intervention (Bremer et al., 2015). Of these reports, the current study reports the lowest dosage, yielding trending results for ball skills and total, but not locomotor scores. Thus, this study adds knowledge that the minimal instruction time for this population lies somewhere above 10 h.

It should be acknowledged that the minimal instructional time question is addressed here independent of pedagogy, which is of utmost importance. FMS interventions to date have employed many pedagogies and drawn upon many different curricula. This consideration is not trivial, and this area should continue to be fervently investigated. However, the paucity of research and practical demands in this area necessitates a simplified discussion first: how much instructional time is necessary to make a difference in FMS, regardless of instructional methods?

The time*group interactions in this study show large effect sizes for ball skills and total scores that were not statistically significant, but approached significance. The interaction for locomotor scores did not approach significance and was of small effect size, suggesting a differential influence of the intervention. Ball skills may require less instruction because, unlike locomotor skills, their equipment communicates the purpose of the movement. This “purpose” information may provide an advantage for young learners with high severity ASD, of which this sample is largely comprised. Interestingly, a similar study previously found a similar differential influence upon locomotor vs. ball skills (Bremer et al., 2015). APE teachers may want to consider this when planning instructional time between these domains; locomotor skill improvement may be slower.

This is the first early FMS intervention to our knowledge that was designed and implemented in the EIBI setting. The implementors in this case were not educated in APE techniques, nor were they intimately familiar with the correct form of the FMS being taught. It is therefore likely that trained APE teachers might be more effective and therefore require less time to influence the FMS of similar participants. In addition, no positive data is presented, instead the current study is an analysis of mid-point results of a larger intervention from which data is forthcoming. Because of this, the current study does not include information on whether the children would have ever learned the motor skills if given enough instructional time. A further limitation is that only two subsets of motor skills were tested due to practical constraints of implementing a study within a functioning clinical center. In fact, one of these subsets (locomotor skills) is already known to be difficult to improve in children with ASD (Bremer et al., 2015). If other components of motor performance could have been tested, results could have been richer. The results approach significance with large effect sizes already at the mid-point, even with these implementors. The results of this study are not necessarily generalizable to every preschooler with ASD, as this sample was comprised of children with relatively severe ASD. Overall, this study adds to a growing body of literature examining methods for impacting the motor development of children with ASD.

## Implications

Ten hours of direct FMS instruction (at mid-intervention) was not enough for the improvement of FMS in preschoolers with ASD. This amount and type of treatment approaches sufficiency for alteration of ball skills, but not locomotor skills.

## Data Availability Statement

The datasets generated for this study are available on request to the corresponding author.

## Ethics Statement

The studies involving human participants were reviewed and approved by the Michigan State University Human Research Protection Program ethics board. Written informed consent to participate in this study was provided by the participants’ legal guardian/next of kin.

## Author Contributions

IF-K and JH conceived the study design. IF-K collected data, analyzed the data, and wrote the manuscript. JH collected data and reviewed the manuscript.

## Conflict of Interest

The authors declare that the research was conducted in the absence of any commercial or financial relationships that could be construed as a potential conflict of interest.
